# Prostate Cancer Diagnosis, Treatment and Outcomes in Patients with Previous or Synchronous Colorectal Cancer: A Systematic Review of Published Evidence

**DOI:** 10.3390/diagnostics12061475

**Published:** 2022-06-15

**Authors:** Giuseppe Celentano, Massimiliano Creta, Luigi Napolitano, Marco Abate, Roberto La Rocca, Marco Capece, Claudia Mirone, Simone Morra, Francesco Di Bello, Luigi Cirillo, Francesco Mangiapia, Gianluigi Califano, Claudia Collà Ruvolo, Caterina Sagnelli, Antonello Sica, Armando Calogero, Fabrizio Iacono, Ferdinando Fusco, Vincenzo Mirone, Nicola Longo

**Affiliations:** 1 Department of Neurosciences, Reproductive Sciences and Odontostomatology, University of Naples Federico II, 80130 Naples, Italy; dr.giuseppecelentano@gmail.com (G.C.); luiginap89@gmail.com (L.N.); marcoabate5@gmail.com (M.A.); roberto.larocca@unina.it (R.L.R.); drmarcocapece@gmail.com (M.C.); simonemorra@outlook.com (S.M.); fran.dibello12@gmail.com (F.D.B.); cirilloluigi22@gmail.com (L.C.); mangiapippo@libero.it (F.M.); gianl.califano2@gmail.com (G.C.); c.collaruvolo@gmail.com (C.C.R.); fiacon@tin.it (F.I.); mirone@unina.it (V.M.); nicolalongo20@yahoo.it (N.L.); 2 Multidisciplinary Department of Medical, Surgical and Dental Sciences, University of Campania “Luigi Vanvitelli”, 80123 Naples, Italy; claudiamirone@outlook.it; 3 Department of Mental Health and Public Medicine, University of Campania “Luigi Vanvitelli”, 80131 Naples, Italy; caterina.sagnelli@unicampania.it; 4 Department of Precision Medicine, University of Campania “Luigi Vanvitelli”, 80123 Naples, Italy; antonello.sica@fastwebnet.it; 5 Department of Advanced Biomedical Sciences, University of Naples Federico II, 80130 Naples, Italy; armando.calogero2@unina.it; 6 Urology Unit, Department of Woman, Child and General and Specialized Surgery, University of Campania “Luigi Vanvitelli”, 80131 Naples, Italy; ferdinando.fusco@unicampania.it

**Keywords:** prostate cancer, colorectal cancer, synchronous cancer, metachronous cancer

## Abstract

The management of patients with prostate cancer (PCa) and previous or synchronous colorectal cancer (CRC) represents a challenging issue. A systematic review was performed in May 2022 to summarize available evidence about the diagnosis, management, and outcomes of these patients. Twenty-seven studies involving 252 patients were identified. Overall, 163 (64.7%) and 89 (35.3%) patients had synchronous and metachronous PCa and CRC, respectively. In patients with synchronous diseases, PCa treatment involved active surveillance in 1 patient, radical prostatectomy (RP) in 36 patients, radiotherapy (RT) in 60 patients, RP plus RT in 1 patient, proton beam therapy in 1 patient, and cryoablation in 1 patient. In patients with previous CRC treatment, prostate biopsy was mostly performed by transrectal approach (*n =* 24). The trans-perineal and suprapubic approaches were adopted in 12 and 6 cases, respectively. Surgical PCa treatment in these cases involved endoscopic extraperitoneal RP, robot-assisted RP, and not otherwise specified RP in 30, 15, and 2 cases, respectively. Biochemical recurrence rates ranged from 20% to 28%. Non-surgical PCa treatment options included brachytherapy, RT plus androgen deprivation therapy, and RT alone in 23, 2 and 4 patients, respectively. PCa specific survival was reported by one study and was 100%.

## 1. Introduction

Prostate cancer (PCa) and colorectal cancer (CRC) represent two of the most common cancers in males contributing to 15% and 9% of new cancers, respectively [1,2,3,4,5,6]. The coexistence of both tumors has been frequently described. In their study, Enblad et al. found a relative risk of 2.2 for the diagnosis of a second primary PCa within 1 year after the diagnosis of CRC [3]. Currently, there are well established separate guidelines for the management of PCa and CRC patients. However, care standards for the management of patients with synchronous or metachronous PCa and CRC are still lacking, thus representing a diagnostic and therapeutic challenge. From a diagnostic point of view, abdominoperineal resection, often performed for the surgical treatment of CRC cancer, may represent a limitation for conventional digital rectal examination and transrectal prostate biopsy. From a therapeutic point of view, combining curative-intent management for both cancers can be challenging given the anatomic proximity of both malignancies and overlapping toxicity risks to surrounding tissues. Unfortunately, there are not enough data for guiding the multidisciplinary strategy and there is a clinically unmet need in determining the preferred treatment for synchronous or metachronous PCa/CRC [7]. Although in the late 1990s, several case reports have been reported describing varying management approaches for the treatment of synchronous CRC and PCa with favorable outcomes, in recent years, the diagnostic and treatment algorithms for these cancers have significantly evolved [1,2,3,4,8,9,10,11,12]. The aim of the present systematic review is to summarize available evidence about the diagnosis and treatment of PCa in patients with synchronous CRC or a history of CRC. 

## 2. Materials and Methods

The present analysis was conducted and reported according to the general guidelines recommended by the Primary Reporting Items for Systematic Reviews and Meta-Analyses (PRISMA) statement [13]. This protocol was registered in PROSPERO (CRD42022328291).

### 2.1. Literature Search

The search was performed in the Medline (US National Library of Medicine, Bethesda, MD, USA), Scopus (Elsevier, Amsterdam, The Netherlands), and Web of Science Core Collection (Thomson Reuters, Toronto, ON, Canada) databases up to May 2022. The following terms were combined to capture relevant publications: “prostate cancer”, “colorectal cancer”, “rectal cancer”, “colon cancer” “synchronous”, “simultaneous”, and “metachronous”. Reference lists in relevant articles and reviews were also screened for additional studies. Conference abstracts were also considered.

### 2.2. Selection Criteria, Data Collection, and Statistical Analysis

Two authors (L.N. and G.C.) reviewed the records separately and individually to select relevant publications, with any discrepancies resolved by a third author (M.C.). To assess eligibility for the systematic review, PICOS (participants, intervention, comparisons, outcomes, and study type) criteria were used [14]. PICOS criteria were set as follows: Participants—patients with PCa and concurrent of previous history of CRC; Intervention—any therapy for PCa in case of history of CRC, any therapy for CRC and PCa in case of simultaneous CRC and PCa; Comparator—none; Outcome—intraoperative and peri-operative outcomes; Study types—conference abstracts, case reports, case series, retrospective and prospective studies. The following data were extracted from eligible studies: first author, year of publication, study design, sample size, patients’ age, synchronous or metachronous PCa, prostate specific antigen (PSA) levels, Gleason score (GS), PCa stage, CRC location and stage, time from CRC treatment to PCa diagnosis, treatments for PCa and CRC, operative time (OT), complications, estimated blood loss (EBL), length of hospital stay (LOS), catheterization time, incidence of positive surgical margins, need for adjuvant therapy, length of follow up, continence rates, PCa specific survival, overall survival (OS), and PCa recurrence. The quality of included studies was assessed using the Jadad Score or the Methodological Index for Non-Randomized Studies (MINORS) for randomized and non-randomized studies, respectively [15,16]. The JBI tool was adopted to evaluate the quality of case reports and case series [17]. Ethical approval and patient consent were not required for the present study. 

## 3. Results

**Figure 1:**
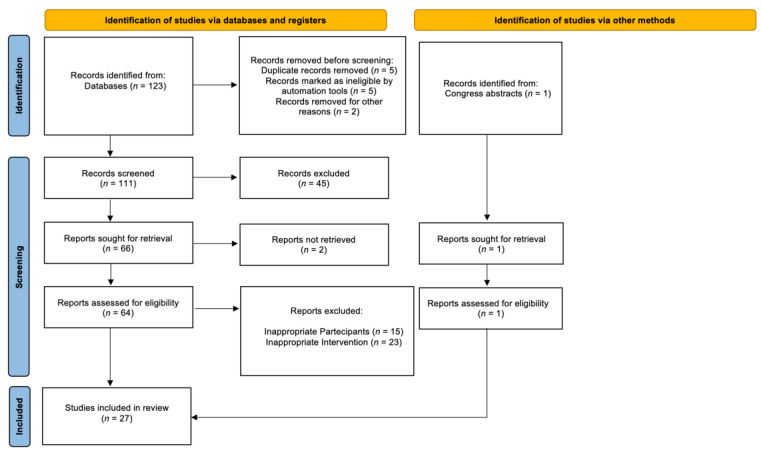
Flow diagram of the systematic review.

The search strategy revealed a total of 124 results. The screening of the titles and abstracts determined 112 papers eligible for inclusion. Further assessment of eligibility, based on the study of the full-text papers, led to the exclusion of 83 papers. Finally, 27 studies involving 252 patients were included in the final analysis (Figure 1) [1,2,7,18,19,20,21,22,23,24,25,26,27,28,29,30,31,32,33,34,35,36,37,38,39,40]. Of these, 19 studies involved only patients with synchronous cancers, 5 studies involved only metachronous PCa patients, and 3 studies involved mixed populations. 

### 3.1. Patient Demographics and Tumor Characteristics

Study characteristics, patients’ demographics and tumors’ features are summarized in Table 1. Mean age ranged from 44 to 84 years. Mean PSA was reported in 24 studies and ranged from 4.1 to 49.6 ng/mL. CRC and PCa were synchronous and metachronous in 163 (64.7%) and 89 (35.3%) patients, respectively. The most common CRC localization was the rectum (alone in 138 patients and in combination with the sigmoid colon in 54 patients). 

### 3.2. Synchronous PCa and CRC

Diagnostic features and treatment outcomes in patients diagnosed with synchronous PCa and CRC are reported in Table 2. Mean PSA values ranged from 4.1 to 49.6 ng/dL. Overall, GS was available in 90 patients (≤6, =7, and ≥8 in 19, 38, and 33 patients, respectively). PCa treatment involved active surveillance in 1 patient, radical prostatectomy (RP) in 36 patients (open radical prostatectomy (ORP), robot-assisted laparoscopic radical prostatectomy (RALP), and not otherwise specified RP in 4, 3, and 29 patients, respectively), radiotherapy (RT) in 60 patients, RP plus RT in 1 patient, proton beam therapy in 1 patient, and cryoablation in 1 patient. Overall, androgen deprivation therapy (ADT) was prescribed in 40 patients. None of the studies involving patients undergoing RP described the incidence of positive surgical margins. Median follow-up ranged from 6 to 60 months. None of the studies reported PCa specific survival.

### 3.3. Metachronous PCa

The details of diagnosis, treatment modalities and outcomes of surgical and non-surgical treatments for PCa in patients with a history of CRC are reported in Table 3 and Table 4.

Mean PSA ranged from 4.8 to 29.5 ng/dL. The technique of prostate biopsy was described in 42 cases, and it was mostly performed by transrectal approach (*n =* 24). The transperineal approach was adopted in 12 cases. In six cases, computed tomography was used to guide suprapubic biopsy. The time between CRC surgery and PCa diagnosis was described in eight studies and ranged from 1 month to 7 years. Overall, GS was available in 71 patients (≤6, =7, and ≥8 in 20, 39, and 12 patients, respectively). Surgical PCa treatment involved endoscopic extraperitoneal radical prostatectomy (EERPE) in 30 patients, RALP in 15 patients, and not otherwise specified RP in 2 cases. Mean OT ranged from 168 to 235 min. Conversion to open surgery was described in three patients undergoing RALP. LOS ranged from 6.0 to 10.8 days and catheterization time ranged from 6.0 to 7.8 days. None of the patients undergoing surgery required adjuvant therapy. None of the studies described the incidence of positive surgical margins. Median follow-up of patients undergoing surgery for PCa ranged from 6.0 to 53.1 months and biochemical recurrence (BCR) rates ranged from 20% to 28%. Non-surgical PCa treatment options included brachytherapy (BT) in 23 patients, radiotherapy (RT) plus ADT in 2, and RT alone in 4 patients. ADT alone was prescribed in five cases. Median follow-up of patients undergoing non-surgical PCa treatment ranged from 18.6 to 85.7 months. PCa specific survival was reported by one study and was 100% at a median follow-up of 85.7 months [26]. 

## 4. Discussion

The incidence of double/multiple primary malignant tumors has risen in the last decades. PCa and CRC represent the most common cancers in males, and they are commonly diagnosed at a mean age of 66 and 65 years, respectively. In recent years, an improvement in the survival of patients diagnosed with these tumors has been observed due to the early detection, the improvement of diagnostic techniques, and the use of new therapies [39]. PCa diagnosis in patients with CRC may be simultaneous or metachronous. Although the coexistence of PCa and CRC has been widely described, the pathophysiology of this association is still under debate. The aggregation of these tumors within families is probably due to a combination of both genetic and environmental factors, with environmental exposures occurring earlier in life being more important. However, further studies are needed to determine the relative contribution of genes and shared environment to the risk of both cancers [41]. Similarly, data concerning the management of these patients are scarce and available guidelines only consider the management of patients with individual conditions [42,43].

Current options with curative intent to manage PCa patients include active surveillance, RP and RT. The choice strongly depends on life expectancy, PCa risk group and patients’ preference. To our knowledge, we performed the first systematic review of studies evaluating the management and outcomes of PCa diagnosis and treatment in patients with synchronous or metachronous CRC. Interestingly, most available data is about patients with synchronous tumours. Of note, this condition is expected to increase in the next years due to improved life expectancy, increased screening programs for both malignancies, and increased use of pelvic magnetic resonance imaging [37]. Therefore, it has been recently suggested to perform CRC screening in men aged ≥ 45 years with newly diagnosed non-metastatic PCa prior to treatment [37]. For synchronous PCa and CRC, there are several potential management alternatives: surgical excision of both tumors using conventional or mini-invasive approaches, excision of the CRC and external beam RT for PCa, external beam RT for both cancers, radio-chemotherapy followed by surgery for CRC combined with ADT, or watchful waiting for PCa [39]. Simultaneous single-session resection of the prostate and rectal cancer has been performed with both open and minimally invasive approaches with acceptable results. In 1999, Klee et al. published their experience with a small series of men who underwent simultaneous non-nerve-sparing radical retropubic prostatectomy and abdominoperineal resection or low anterior resection of the rectum [19]. As underlined by Park et al., simultaneous robotic low anterior resection of the rectum and RP represents a promising minimally invasive surgical option for patients with a simultaneous diagnosis of PCa and rectal cancer [29]. Indeed, the assistance of the robot system can allow higher precision and effectiveness in conducting combined procedures, and both surgical teams can share the same port sites to perform both operations without disturbing each operative strategy [29]. Issues concerning combined surgical procedures that need to be further addressed include the cost-effectiveness and post-operative urinary incontinence [29]. One of the concerns with combined RP and low anterior resection of the rectum is the risk of fistula formation between the bladder and the bowel due to the presence of overlying adjacent anastomoses [19]. Functional outcomes remain an issue of concern. Nerve sparing is considered to be difficult, if not impossible, and might significantly compromise wide resection of the rectal tumors. RT should be considered a treatment option for the simultaneous treatment of both malignancies. Radiation dose escalation represents one of the major concerns. Indeed, PCa disease-related outcomes are improved with radiation dose escalation to levels higher than what is typically given for rectosigmoid cancers. Therefore, complications associated with rectosigmoid surgery such as anastomotic leak, fistula, or strictures can significantly increase [37]. Some authors argue that the prognosis of patients with concomitant CRC and PCa mostly depends on CRC evolution, and that the median follow-up might be too short to evaluate the oncologic outcomes of PCa [7]. Interestingly, the low local CRC recurrence rate observed by some authors raised the question of increased local control due to concurrent RP. However, this association remains correlative rather than conclusive, and further studies are needed to confirm this hypothesis [7]. It is not uncommon that CRC survivors will require a treatment for PCa during their lifetime [40]. It has been reported that approximately one-sixth of men aged ≥ 50 years with > 10 years life expectancy undergoing PCa screening prior to CRC surgery had synchronous PCa [37]. Moreover, screening colonoscopies in men with newly diagnosed PCa identified synchronous CRC in > 3% of men [37]. Most patients with metachronous PCa diagnosis underwent RP, and a minimally invasive approach was adopted in most cases. Overall, OT associated with both EERPE and RALP were higher if compared to published data in patients without history of CRC surgery. However, mean EBL, LOS and catheter duration were within published ranges. It has been reported that one of the major challenges of prostate surgery after previous CRC management is the presence of periprostatic and intra-abdominal adhesions. Periprostatic adhesions may render the identification of the plans challenging, especially during seminal vesicle and endopelvic fascia dissection, and may compromise perioperative and functional outcomes of prostatectomy. Intra-abdominal adhesions have been traditionally regarded as a relative contraindication to minimal-invasive RP [31]. Indeed, history of abdominal surgery is a predictor of adhesions, and autopsy studies revealed that intra-abdominal adhesions appeared in approximately 90% of patients after abdominal surgery, thus leading to the need for extensive adhesiolysis to allow trocar placement with the increased risk of bowel injury [31]. Currently, RALP has become the main surgical option for the treatment of PCa. Technological advances of robot-assisted laparoscopic surgery, such as improved visualization and more controlled finer movements, are typically associated with better dissection, thus potentially capable of reducing challenges associated with prostate surgery in patients with previous major abdominal surgery or radiation [33]. Luciani et al. published one of the most recent series on RALP in patients with a history of CRC surgery [40]. Overall, 14 RALP were performed after previous colorectal surgery (resection of the left colon (*n* = 6), resection of the right colon (*n* = 4), resection of the rectum (*n* = 4)) after an interval of 5 years. Interestingly, most prostatectomies were completed robotically (*n* = 11), median blood loss was low, no major postoperative complication was reported, and hospital stay was in line with standards (all patients removed the catheter and were discharged on postoperative day 6). Eleven (78%) patients were continent at 1 year. Four patients with non-organ-confined disease (pT3a-b N0-1 Gleason score 8 or higher) experienced biochemical persistence or recurrence and underwent adjuvant RT during a median follow-up of 3.5 years [40]. Alternative options include RT and BT. The treatment of double/multiple primary malignant tumors is a challenge for the medical and surgical teams. Issues to be addressed include patients’ age, comorbidities, tumors’ risk category, preference of patients, and the equipment of the hospital [39]. The present review represents the first attempt to summarize in a systematic fashion available data related to the diagnosis, therapy, and outcomes of PCa in patients with previous or concomitant CRC, an often-neglected subset of patients whose epidemiological relevance is expected to increase in the next few years. Available data show that the management of these patients can be safely achieved with curative intent in a substantial percentage of patients. However, this review is not devoid of limitations. They mostly reflect the drawbacks related to the literature on this topic. Most series are small, retrospective, with short follow-up, and derive from a single institution. Moreover, most studies have an uncontrolled design. Furthermore, significant heterogeneity exists in terms of patient characteristics, treatment protocols, and outcomes assessed. Treatment-related complications, one of the major surgical concerns in these patients, are often reported in a non-standardized fashion. In everyday clinical practice, it is advisable to plan the diagnostic work-up and therapeutical management in an individualized fashion and within a multidisciplinary team, taking into account the particularities of the case and the expertise of the treating center. 

## 5. Conclusions

Despite the growing epidemiological relevance of double/multiple primary malignant tumors, current evidence about the diagnosis, management, and outcomes of synchronous or metachronous PCa in CRC patients remains suboptimal, deriving only from retrospective small series and case reports. In patients with synchronous diseases, single-session RP, and CRC surgery, as well as RT with or without ADT, represent treatment options with promising short-term oncologic PCa outcomes. In patients with a history of CRC who receive a diagnosis of PCa, minimally invasive RP techniques have been investigated with promising perioperative outcomes. RT with or without ADT represents a potential alternative. However, the level of evidence is still suboptimal, and results from well conducted, multi-institutional series with adequate follow-up are required.

## Figures and Tables

**Table 1:** Study characteristics and patients’ clinic demographic data.

Author, yr	Study Design	Quality Score	Sample Size (*n*)	Mean Age, Years	S/M, *n*	Mean PSA ng/mL	GS, *n*	PCa Stage, *n*	CRC Location, *n*	CRC Stage, *n*	Time from CRC Surgery to PCa Diagnosis
Baur, 1997 [18]	CR	Mo	1	65.0	S, 1	n/a	n/a	T3N0M0, 1	n/a	T2N0M0, 1	n/a
Klee, 1999 [19]	CS	Mo	3	62.0	S, 3	7.0	6, 2 7, 1	pT2aN0Mx, 1 pT2cN0Mx, 1 pT3bN0Mx, 1	Re, 3	A *, 1 B *, 1 n/a, 1	n/a
Terris, 2001 [2]	CS	Hi	3	n/a	S, 3	16.1	6, 1 (3 + 4), 1 8, 1	T1c,1 T2a, 1 T3, 1	n/a	n/a	n/a
Siu, 2001 [20]	CS	Mo	2	72.5	S, 2	8.4	6, 1 (4 + 3), 1	cT1cNxMx, 1 cT2aNxM0, 1	Re, 2	T3, 1 T4,1	n/a
Colonias, 2005 [21]	CR	Mo	1	58.0	S, 1	32.0	6, 1	T1cN0Mo, 1	Re,1	pT3N1M0, 1	n/a
Koutrouvelis, 2005 [22]	R	12	5	64.0	M, 5	8.8	6, 3 7, 1 8, 1	T1c, 2 T2b, 1 T3a, 1 T3b, 1	Re, 5	n/a	n/a
Jabbari, 2009 [23]	CS	Mo	6	66.5	M, 6	6.8	6, 2 (3 + 4), 2 8, 2	T1cN0M0, 3 T2N0M0, 2 T2bN0M0, 1	n/a, 6	n/a	>5 yr
Ayhan, 2011 [24]	CR	Lo	1	84.0	S, 1	10	8, 1	T3aN0M0,1	Re, 1	T3N0, 1	n/a
Lin, 2011 [25]	CS	Mo	3	66.0	S, 3	49.6	(2 + 2), 1 (2 + 3), 1 (3 + 2), 1	T2N2M0, 1 T3N0M0,1 T3N1M0, 1	Mid Re, 3	B *, 2 C *, 1	n/a
Sharp, 2012 [26]	R	14	18	65.1	M, 12	6.8	6, 5 (3 + 4), 3 (4 + 3), 3 (4 + 4), 1	T1c, 10 T2a, 2	RS, 2 Sigmoid, 3 RC, 1 Re, 1 Multiple, 1 n/a, 4	TXN0M0, 2 TXNXMX, 2 TisN0M0, 2 T1N0M0, 1 T1N1M0, 1 T2N0M0, 1 T3N0M0, 2 T3N1M0, 1	n/a
				63.0	S, 6	7.8	(3 + 4), 3 (4 + 3), 2 (4 + 4), 1	T1c, 3 T2a, 2 TX, 1	RS, 1 Sigmoid, 2 Re, 2 RC, 1	TisN0M0,1 T1N0M0, 3 T3N0M0, 1	n/a
Kavanagh, 2012 [1]	R	11	12	70.8	S, 9	21.4	n/a	n/a	Low Re, 12	n/a	n/a
				62.3	M, 3						>3 mo
Sturludottir, 2015 [27]	R	16	29	73.8	S, 29	17.0	n/a	n/a	Low Re, 8 Mid Re, 11 High Re, 7	T2, 3 T3, 12 T2/T3, 1 T4, 3 T3/T4, 1 N1, 13	n/a
Lavan, 2015 [6]	R	13	10	68.0	S, 10	13.0	5, 1 6, 2 (3 + 4), 3 9, 4	TxN0, 1 T1cN0, 2 T1cNx, 1 T2cN0, 1 T2cNx, 1 T3bNx, 2 T3bN1, 2	Low Re, 2 Mid Re, 5 High Re, 3	T2N0, 2 T2N1, 1 T3N0, 4 T3N1, 1 T4bN0, 1	n/a
Kamiyama, 2015 [28]	CR	Mo	1	74.0	S, 1	n/a	n/a	pT2aN0M0, 1	Re, 1	pTisN0M0, 1	n/a
Park, 2016 [29]	CR	Mo	1	64.0	S, 1	4.1	(3 + 4), 1	pT2cN0, 1	Re, 1	pT3N0M0, 1	n/a
Owens, 2017 [30]	CS	Lo	6	73.5	S, 6	12.9	7, 3 8, 2 9, 1	T2cn0, 1 T3n0, 3 T3n1, 1 T3bn1, 1	n/a	T1N0, 1 T3N0, 2 T3N1, 2 T4N0, 1	n/a
Liu, 2017 [31]	CS	Mo	30	66.3	M, 30	13.4	6, 8 7, 20 8, 1 10, 1	pT2a, 2 pT2b, 8 pT2c, 14 pT3a, 2 pT3b, 1	Re, 30	pT1, 4 pT2, 26	6.3 yr
Gys, 2017 [32]	CR	Lo	1	61.0	S, 1	30.2	8/9, 1	T2cN1M0, 1	Sigmoid, 1	pT3N2bM0, 1	n/a
Basatac, 2018 [33]	CR	Lo	1	74.0	M, 1	4.8	8, 1	n/a	Re, 1	n/a	5 yr
Villegas-Otiniano, 2018 [34]	CR	Mo	1	77.0	S, 1	24.8	(3 + 4), 1	n/a	Re, 1	n/a	n/a
Doussot, 2020 [7]	R	16	25	71.0	S, 25	n/a	n/a	n/a	Mid Re, 12 Low Re, 13	T0, 2 T1-T2, 10 T3, 11 T4, 2 N0, 17 N1, 7 N2, 1 M0, 24 M1, 1	n/a
Tey, 2020 [35]	CR	Mo	1	69.0	S, 1	20.0	(3 + 4), 1	T2bN0M0, 1	Re, 1	cT3N1M0, 1	n/a
Kojima, 2020 [36]	CR	Lo	1	44.0	S, 1	n/a	n/a	n/a	Low Re, 1	T4bN1M0, 1	n/a
Jacobs, 2020 [37]	R	13	54	67.0	S, 54	10.8	6, 8 (3 + 4), 8 (4 + 3), 10 8–9, 21	T1b-T1c, 16 T2a-T2c, 17 T3a-T3b, 9 N0, 46 N1, 2 n/a, 12 pT4aN2a, 1 M0, 46 M1, 2	RS, 54	T1, 14 T2-3, 31 T4, 9	n/a
Chiang, 2021 [38]	CR	Lo	1	65.0	S, 1	14.1	(4 + 3), 1	cT2aN0M0, 1	High Re, 1	cT2N2bM0	n/a
Dema, 2021 [39]	R	13	21	67.3	S, 3	26.2	(3 + 4), 2 (4 + 5), 1	T1b, 1 pT3a, 1 pT4N1, 1	Re, 2 LC, 1	pT4aN1bM1c, 1 PT1N1a, 1 pT2N0, 1	n/a
				71.8	M, 18	29.5	(2 + 4), 1 (3 + 3), 1 (3 + 4), 9 (4 + 3), 1 (4 + 4), 1 (4 + 5), 2 (5 + 4), 1 (5 + 5), 1 n/a, 1	T2c, 1 T1b, 2 pT3b, 1 pT4, 2 n/a, 12	Re, 4 RC, 4 LC, 11	pT1, 1 pT2N0, 4 pT3N0, 6 pT3N2a, 1 pT4aN0, 1 pT4aN2a, 1 pT4aN1b,2 pT4aN2aM1c, 1	64.5 mo
Luciani, 2021 [40]	R	12	14	64.0	M, 14	n/a	n/a	n/a	LC, 6 RC, 4 Re, 4	pT0-T2N0, 5 pT3N0-1, 3 Benign, 4 n/a, 2	5 yr

CR: case report; CRC: colorectal cancer; CS: case series; GS: Gleason score; Hi: high; LC: left colon; Lo: low; M: metachronous; Mo: moderate; n/a: not applicable; P: prospective; PCa: prostate cancer; R: retrospective; RC: right colon; Re: rectum; RS: rectosigmoid; S: synchronous; yr: years; *: Dukes stage.

**Table 2:** Diagnosis, treatment and outcomes in patients with synchronous PCa and CRC.

Author, yr	PCa Suspect Type, *n*	PCa Diagnosis Type, *n*	PCa Therapy Type, *n*	CRC Therapy Type, *n*	OT, Min, Mean	Complications Type, *n*	Continence Rates, % (mo)	EBL, mL, Mean	Catheter Duration, Days, Mean	LOS, Days, Mean	Fu, mo, Median	PCa Recurrence	OS, %
Baur, 1997 [18]	n/a	NOS Biopsy, 1	ORP, 1	PRR, 1	n/a	n/a	100 (24)	n/a	n/a	n/a	24.0	n/a	100
Klee, 1999 [19]	DRE, 2 PSA, 3	NOS Biopsy, 3	ORP, 3	APR, 2 LAR, 1	n/a	Bowel obstruction, 1 Rectal and bladder neck stricture, 1	100 (6)	1000.0	14.0	10.0	12.0	0	100
Siu, 2001 [20]	PSA, 2	TRB, 2	RT, 2	CT, 1 RT, 1	n/a	n/a	n/a	n/a	n/a	n/a	24.0	0	100
Colonias, 2005 [21]	PSA, 1	TRB, 1	RT + ADT + RP, 1	CT + RT + 5FU + PS, 1	n/a	0	100 (14)	n/a	n/a	n/a	14.0	0	100
Lin, 2011 [25]	DRE, 3	TRB, 3	RRP, 3	LAR, 2 APR, 1	n/a	0	n/a	n/a	n/a	n/a	22.6	n/a	66
Kavanagh, 2012 [1]	DRE, 7 MRI, 2	n/a	ADT, 3 AS, 1 RT, 4 RP, 1	CT, 1 AR, 1 RT, 1 APR, 1 PE, 1 PE, 1 RT + AR, 1 Palliation, 1	n/a	Wound infection, 2 Intra-abdominal collection requiring radiological drainage, 1 Foot drop, 1	n/a	n/a	n/a	n/a	8.5	BM, 1	25
										33.0	26.4		60
Sharp, 2012 [26]	n/a	n/a	RP, 6	n/a	n/a	n/a	n/a	n/a	n/a	n/a	n/a	n/a	100
Sturludottir, 2015 [27]	MRI, 14 DRE, 6 PSA, 2 n/a, 2 LUTS, 1 BM, 1 Incidental, 1	n/a	n/a	n/a	n/a	n/a	n/a	n/a	n/a	n/a	n/a	0	n/a
Kamiyama, 2015 [28]	n/a	n/a	RALP, 1	CT + RLAP, 1	545.0	n/a	n/a	170.0	n/a	17.0	n/a	0	n/a
Lavan, 2015 [6]	n/a	TRB/TPB, 10	RT, 10 ADT, 4	PE, 1 AR, 7 APR, 1 5FU, 9 CT, 8	n/a	Proctitis, 2 Frequency/urgency, 4 Hesitancy, 1 Cystitis, 1 Diarrhoea, 1 Nocturia, 1	n/a	n/a	n/a	n/a	26.0	BM, 2 Castrate resistant disease, 1	90
Park, 2016 [29]	n/a	n/a	RALP, 1	RLAR, 1	360.0	0	100 (5)	350.0	7.0	10.0	n/a	0	100
Owens, 2017 [30]	n/a	n/a	ADT + RT, 6	RT, 6 CT, 5 TAE, 1	n/a	Fatigue, 5 Pelvic pain, 4 Diarrhoea, 3 Constipation, 1 Anorexia, 1 Palmoplantar erythema, 1	n/a	n/a	n/a	n/a	n/a	n/a	n/a
Gys, 2017 [32]	MRI, 1	n/a	RALP, 1	TAE, 1	720.0	Bleeding peptic ulcer, 1	100 (1)	450.0	21.0	23.0	6.0	0	100
Villegas-Otiniano, 2018 [34]	PSA, 1	n/a	RT + ADT, 1	CT + RT, 1	n/a	Perianal abscess, 1	n/a	n/a	n/a	n/a	60.0	0	100
Tey, 2020 [35]	DRE, 1	TRB, 1	ADT + RT + BT, 1	RT + CT + LAR, 1	n/a	Cystitis, 1 Fatigue, 1	n/a	n/a	n/a	n/a	12.0	0	100
Jacobs, 2020 [37]	PSA, 54	n/a	RP, 17 RT, 10 ADT, 23 CA, 1 TURP, 1 n/a, 1	APR, 10 LAR, 24 TAE, 7 PE, 1 RT, 28	n/a	Pelvic/femur fracture, 1 Fistula, 4 Urosepsis, n/a Erectile dysfunction, 6 Death, 2	n/a	n/a	n/a	n/a	43.0	Biochemical recurrence, 12 Castrate resistance, 3 Metastases, 4	89.7
Doussot, 2020 [7]	n/a	n/a	RT, 24 RP, 1 n/a, 1	AR, 15 APR, 4 AR, 1 PE, 5	n/a	Death, 3 Anastomotic leak, 2	n/a	n/a	n/a	13.0	31.3	0	76
Kojima, 2020 [36]	n/a	n/a	RALP, 1	CT + robotic LAR, 1	949.0	0	n/a	290.0	n/a	n/a	n/a	0	100
Chiang, 2021 [38]	MRI, 1	NOS biopsy, 1	PBT+ RT + ADT, 1	RLAR + CT, 1	n/a	Bowel urgency and frequency, 1 Urinary leakage, 1 Erectile dysfunction, 1	n/a	n/a	n/a	n/a	37.0	n/a	100
Dema, 2021 [39]	PSA, 2 n/a, 1	NOS biopsy, 1 TURP, 1 Partial prostatectomy, 1	RP + RT, 1 n/a, 2	Hartman, 1 LAR, 1 APR, 1	n/a	n/a	n/a	n/a	n/a	n/a	n/a	n/a	n/a

5FU: 5-fluouracil; ADT: androgen deprivation therapy; APR: abdominoperineal resection; AR: anterior resection; AS: active surveillance; BCR: biochemical recurrence; BM: bone metastases; BT: brachytherapy; CA: cryoablation; CT: chemotherapy; DRE: digital rectal examination; EBL: estimated blood loss; Fu: follow-up; LAR: low anterior resection; LOS: length of stay; LUTS: lower urinary tract symptoms; mo: months; MRI: magnetic resonance imaging; NOS: not otherwise specified; ORP: open radical prostatectomy; OS: overall survival; OT: operative time; n/a: not applicable; PBT: proton beam therapy; PCa: prostate cancer; PE: pelvic exenteration; PRR: partial rectal resection; PS: proctosigmoidectomy; RALP: robot-assisted radical prostatectomy; RLAR: robot-assisted low anterior resection; RP: radical prostatectomy; RT: radiotherapy; TAE: trans-anal excision; TRB: transrectal biopsy; TPB: transperineal biopsy; TURP: transurethral resection of prostate; yr: years.

**Table 3:** Diagnosis, management, and outcomes of surgery for PCa in patients with a history of CRC.

Author, yr	PCa Suspect Type, *n*	Prostate Biopsy, Type, *n*	PCa Surgical Treatment Type, *n*	OT, Min, Mean	EBL, mL, Mean	Intraoperative Complications Type, *n*	Postoperative Complications Type, *n*	LOS Days, Mean	Catheter Duration, Days, Mean	Fu, mo, Median	Continence Rates, % (mo)	OS %	BCR, *n* (%)	Savage Therapy, *n*
Liu, 2017 [31]	n/a	TRB, 24 TPB, 6	EERPE, 30	168.0	195.0	n/a	Lymphocele, 1	10.8	7.8	53.1	86.7 (26)	n/a	6 (20)	n/a
Basatac, 2018 [33]	PSA + MRI, 1	SPB, 1	RALP, 1	181.0	150.0	0	0	8.0	n/a	6.0	100 (3)	100	n/a	n/a
Luciani, 2021 [40]	PSA, 14	NOS, 14	RALP, 14	235.0	450.0	Open conversion, 3 Ileal repair, 2	Anemia, 2 Persistent drain output, 1 UTI, 1	6.0	6.0	41.0	78 (12)	n/a	4 (28)	RT, 4

BCR: biochemical recurrence; CRC: colorectal cancer; CT: chemotherapy; EBL: estimated blood loss; EERPE: endoscopic extraperitoneal radical prostatectomy; Fu: follow-up duration; LOS: length of hospital stay; mo: months; MRI: magnetic resonance imaging; n/a: not applicable; NOS: not otherwise specified; OS: overall survival; OT: operative time; PCa: prostate cancer; RALP: robot-assisted radical prostatectomy; RT: radiotherapy; SPB: suprapubic biopsy; TPB: transperineal biopsy; TRB: transrectal biopsy; UTI: urinary tract infections; yr: years.

**Table 4:** Diagnosis, management, and outcomes of non-surgical treatments for PCa in patients with a history of CRC.

Author, yr	PCa Suspect Type, *n*	PCa Diagnosis Type, *n*	PCa Treatment Type, *n*	Radiation Dose, Gy, *n*	Complications Type, *n*	Fu mo Median	BCR *n* (%)	OS %	PCa CSS %
Koutrouvelis, 2005 [22]	PSA, 5	SP, 5	BT, 5	144, 5	Urinary retention and ureteric stent placement, 1	18.6	n/a	100	n/a
Jabbari, 2009 [23]	PSA, 6	TP, 6	HDR BT, 6	36, 5 24, 1	Urethral stricture, 1 LUTS, 4	26.0	1 (16.6)	n/a	n/a
Sharp, 2012 [26]	n/a	n/a	BT, 12	145, 12	n/a	85.7	n/a	50	100
Kavanagh, 2012 [1]	n/a	n/a	ADT, 2 EBRT + ADT, 1	74, 3	0	26.4	n/a	33	n/a
Dema, 2021 [39]	n/a	NOS biopsy, 13 TURP, 4 n/a, 1	RT + ADT, 1 RP + RT, 1 RP, 1 RP + RT, 1 ADT, 3 RT, 4 n/a, 8	n/a	n/a	n/a	n/a	n/a	n/a

ADT: androgen deprivation therapy; BCR: biochemical recurrence; BT: brachytherapy; CRC: colorectal cancer; CSS: cancer specific survival; EBRT: external beam radiotherapy; Fu: follow-up duration; LUTS: lower urinary tract symptoms; mo: months; n/a: not applicable; NOS: not otherwise specified; OS: overall survival; PCa: prostate cancer; RT: radiotherapy; SPB: suprapubic biopsy; TPB: transperineal biopsy; TURP: transurethral resection of prostate; yr: years.

## Data Availability

Not applicable.
